# The genome sequence of the Atlantic mackerel, *Scomber scombrus *Linnaeus, 1758

**DOI:** 10.12688/wellcomeopenres.23186.1

**Published:** 2024-10-17

**Authors:** Mitchell Brennan, Kimberly Bird, Birthe Zancker, Vengamanaidu Modepali, Patrick Adkins, Marine Biological Association Genome Acquisition Lab, Darwin Tree of Life Barcoding collective, Wellcome Sanger Institute Tree of Life Management, Samples and Laboratory team, Wellcome Sanger Institute Scientific Operations: Sequencing Operations, Wellcome Sanger Institute Tree of Life Core Informatics team, Tree of Life Core Informatics collective, Darwin Tree of Life Consortium

**Affiliations:** 1 The Marine Biological Association, Plymouth, England, UK

**Keywords:** Scomber scombrus, Atlantic mackerel, genome sequence, chromosomal, Scombriformes

## Abstract

We present a genome assembly from an individual *Scomber scombrus* (the Atlantic mackerel; Chordata; Actinopteri; Scombriformes; Scombridae). The genome sequence has a total length of 764.10 megabases. Most of the assembly is scaffolded into 24 chromosomal pseudomolecules. The mitochondrial genome has also been assembled and is 16.56 kilobases in length.

## Species taxonomy

Eukaryota; Opisthokonta; Metazoa; Eumetazoa; Bilateria; Deuterostomia; Chordata; Craniata; Vertebrata; Gnathostomata; Teleostomi; Euteleostomi; Actinopterygii; Actinopteri; Neopterygii; Teleostei; Osteoglossocephalai; Clupeocephala; Euteleosteomorpha; Neoteleostei; Eurypterygia; Ctenosquamata; Acanthomorphata; Euacanthomorphacea; Percomorphaceae; Pelagiaria; Scombriformes; Scombridae; Scombrinae; Scombrini; *Scomber*; *Scomber scombru*s Linnaeus, 1758 (NCBI:txid13677).

## Background

Mackerels are a group of migratory, marine, coastal-pelagic fish in the family Scombridae ( Blaber, 1990). The genus *Scomber* includes four species: *S. scombrus*, *S. japonicus*, *S. australasicus* and *S. colias*. The Atlantic mackerel, *Scomber scombrus*, Linnaeus, 1758, is a highly abundant and widely distributed migratory fish species found across the North Atlantic and adjacent seas, including the Baltic Sea and in the western Atlantic, they are found from Labrador to North Carolina ( Blaber, 1990; Miller & ICES, 2013). These long-distance migratory species undertake seasonal migrations of over 2000 km ( Moores *et al.*, 1975; Parsons & Moores, 1973). Atlantic mackerel spawn from April to May in the Mid-Atlantic Bight, and from June to July in the Gulf of the St Lawrence, with small females laying around 280,000 eggs and larger females laying around 2 million eggs in batches between 5 and 7 times during a spawning season. These fish can live up to 20 years and spend their entire life cycle in the pelagic zone. Eggs and larvae are typically found over the continental shelf, at depths ranging from the surface to 100 m, or near the coast ( Alvarez & Chifflet, 2012). Atlantic mackerel occupies a critical ecological niche, feeding on a variety of zooplankton, phytoplankton, pelagic larval, squid, some fish, and ascidians ( Engelhard & others, 2013; Pinnegar *et al.*, 2015). They are also an essential food source for larger pelagic fish and marine mammals, playing a crucial role in the marine food chain.

Besides its ecological importance, Atlantic mackerel also supports important commercial fisheries for several countries throughout its distribution range ( Collette & Nauen, 1983). The species is widely consumed as a significant source of omega-3 fatty acids, which are in high demand and predominantly derived from fish oil ( Jacobsen *et al.*, 2013). Due to its ecological and economic importance, Atlantic mackerel has been the focus of several recent studies focusing on different aspects of its fisheries and biology ( Gíslason & others, 2020; Stroganov & others, 2023). Despite its ecological and economic importance, the population size of Atlantic mackerel has recently declined due to climate change affecting optimal habitat conditions and temperature-dependent hatching rates, putting the species’ genetic diversity at stake. Genomic resources for the species are still limited, and better understanding of Atlantic mackerel genomes could be invaluable for conservation efforts.

## Genome sequence report

**Figure 1.**
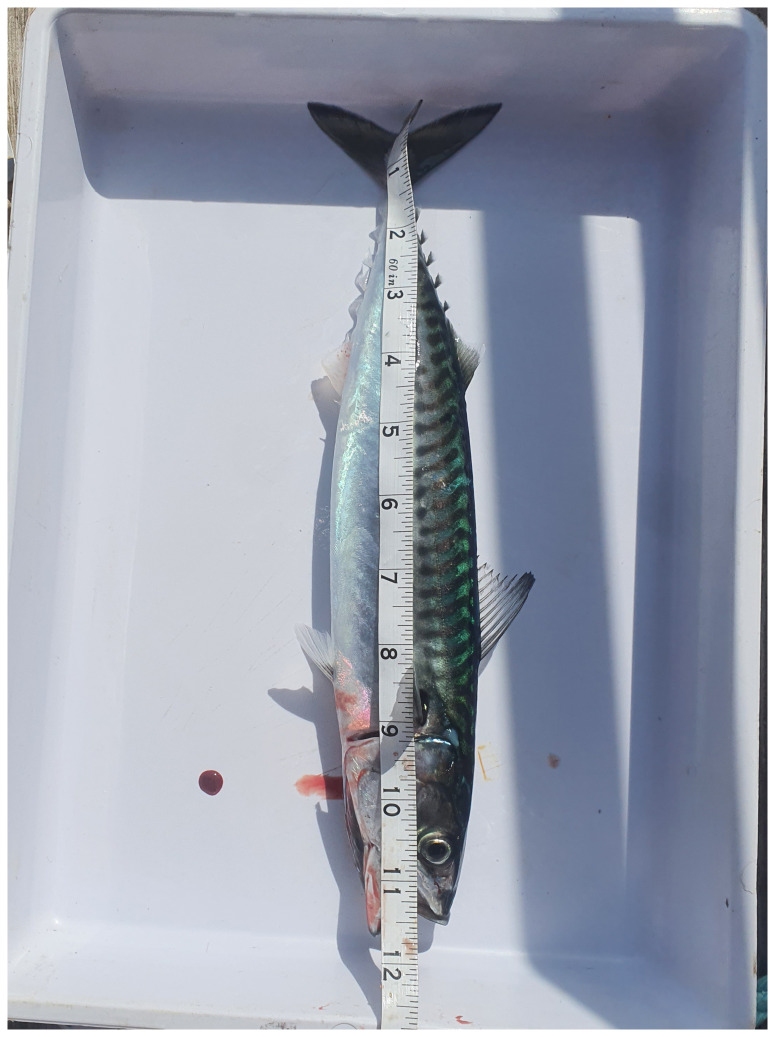
Photograph of the *Scomber scombrus* (fScoSco1) specimen used for genome sequencing.

The genome of an adult *Scomber scombrus* ( Figure 1) was sequenced using Pacific Biosciences single-molecule HiFi long reads, generating a total of 19.92 Gb (gigabases) from 2.15 million reads, providing approximately 29-fold coverage. Primary assembly contigs were scaffolded with chromosome conformation Hi-C data, which produced 106.96 Gb from 708.31 million reads, yielding an approximate coverage of 140-fold. Information about specimens and sequencing is summarised in Table 1.

**Table 1.** Specimen and sequencing data for *Scomber scombrus*.

Project information			
**Study title**	Scomber scombrus (Atlantic mackerel)		
**Umbrella BioProject**	PRJEB69500		
**Species**	*Scomber scombrus*		
**BioSample**	SAMEA110450232		
**NCBI taxonomy ID**	13677		
Specimen information			
**Technology**	**ToLID**	**BioSample accession**	**Organism part**
**PacBio long read sequencing**	fScoSco1	SAMEA110451152	Gill
**Hi-C sequencing**	fScoSco1	SAMEA110451157	Gill
**RNA sequencing**	fScoSco1	SAMEA110451152	Gill
Sequencing information			
**Platform**	**Run accession**	**Read count**	**Base count (Gb)**
**Hi-C Illumina NovaSeq 6000**	ERR12318580	7.08e+08	106.96
**PacBio Sequel IIe**	ERR12303949	2.15e+06	19.92
**RNA Illumina NovaSeq X**	ERR12765152	6.34e+07	9.58

**Figure 2.**
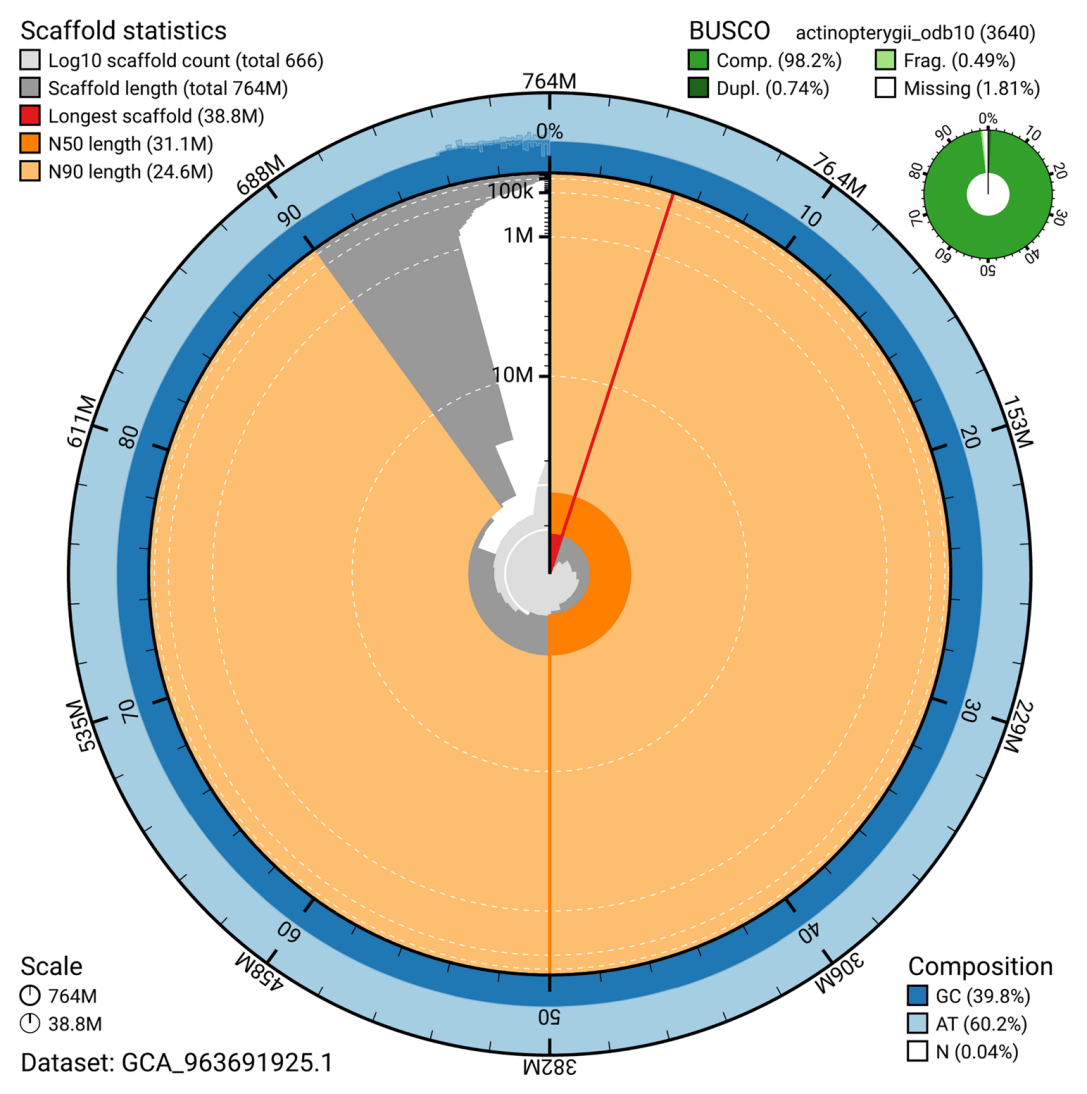
Genome assembly of *Scomber scombrus*, fScoSco1.1: metrics. The BlobToolKit snail plot shows N50 metrics and BUSCO gene completeness. The main plot is divided into 1,000 size-ordered bins around the circumference with each bin representing 0.1% of the 764,157,853 bp assembly. The distribution of scaffold lengths is shown in dark grey with the plot radius scaled to the longest scaffold present in the assembly (38,754,833 bp, shown in red). Orange and pale-orange arcs show the N50 and N90 scaffold lengths (31,095,048 and 24,622,544 bp), respectively. The pale grey spiral shows the cumulative scaffold count on a log scale with white scale lines showing successive orders of magnitude. The blue and pale-blue area around the outside of the plot shows the distribution of GC, AT and N percentages in the same bins as the inner plot. A summary of complete, fragmented, duplicated and missing BUSCO genes in the actinopterygii_odb10 set is shown in the top right. An interactive version of this figure is available at https://blobtoolkit.genomehubs.org/view/GCA_963691925.1/dataset/GCA_963691925.1/snail.

**Figure 3.**
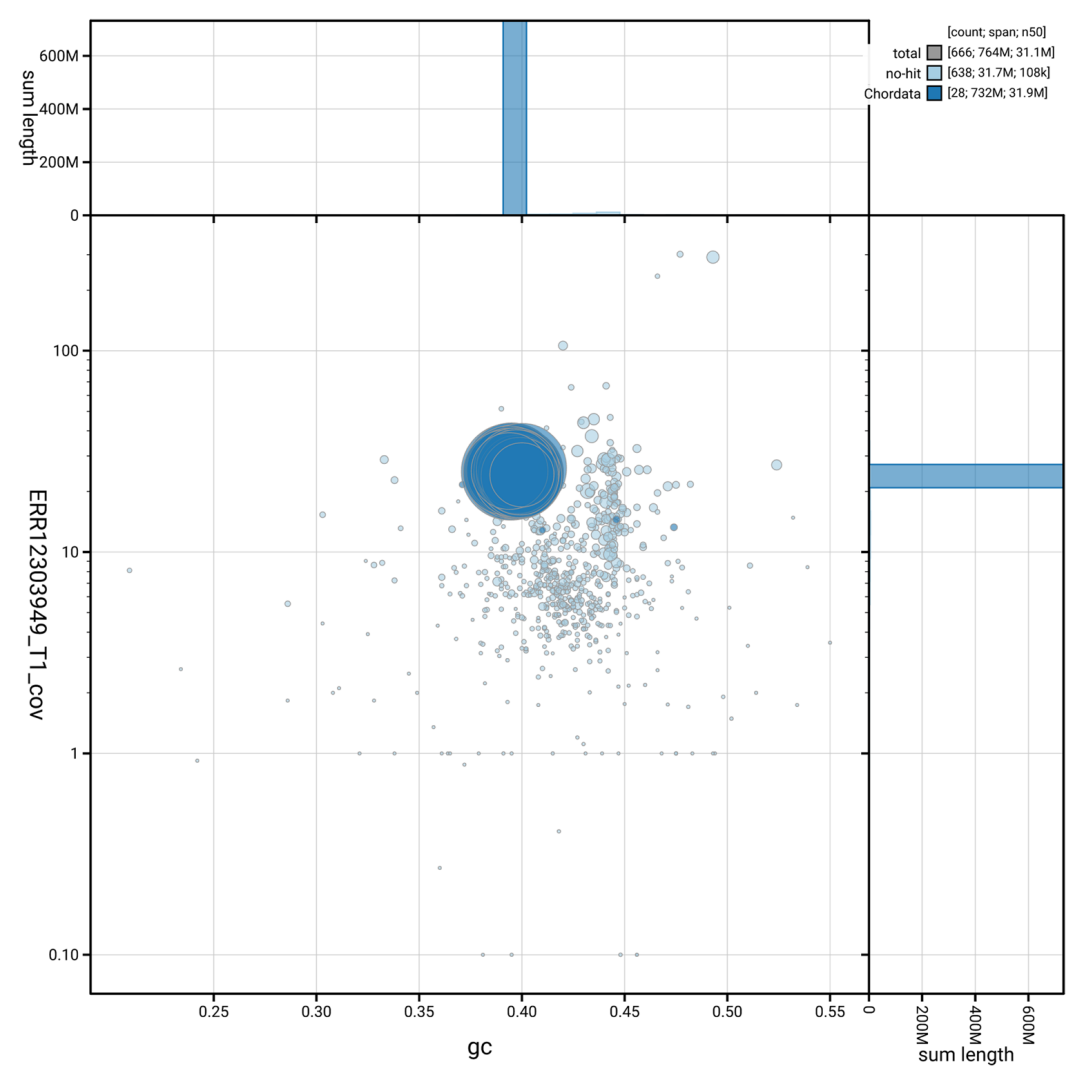
Genome assembly of *Scomber scombrus*, fScoSco1.1: BlobToolKit GC-coverage plot. Sequences are coloured by phylum. Circles are sized in proportion to sequence length. Histograms show the distribution of sequence length sum along each axis. An interactive version of this figure is available at https://blobtoolkit.genomehubs.org/view/GCA_963691925.1/dataset/GCA_963691925.1/blob.

**Figure 4.**
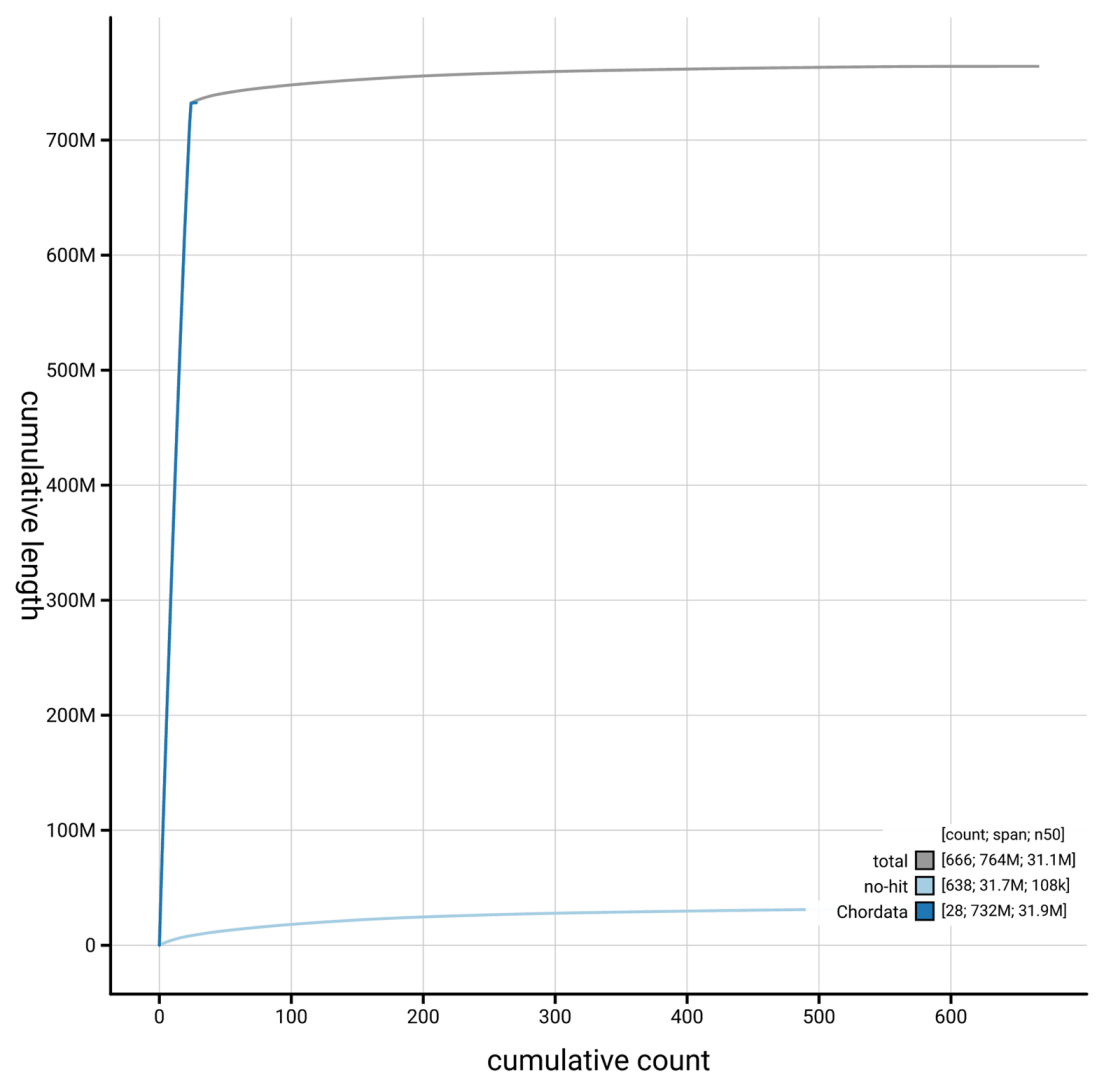
Genome assembly of *Scomber scombrus* fScoSco1.1: BlobToolKit cumulative sequence plot. The grey line shows cumulative length for all sequences. Coloured lines show cumulative lengths of sequences assigned to each phylum using the buscogenes taxrule. An interactive version of this figure is available at https://blobtoolkit.genomehubs.org/view/GCA_963691925.1/dataset/GCA_963691925.1/cumulative.

**Figure 5.**
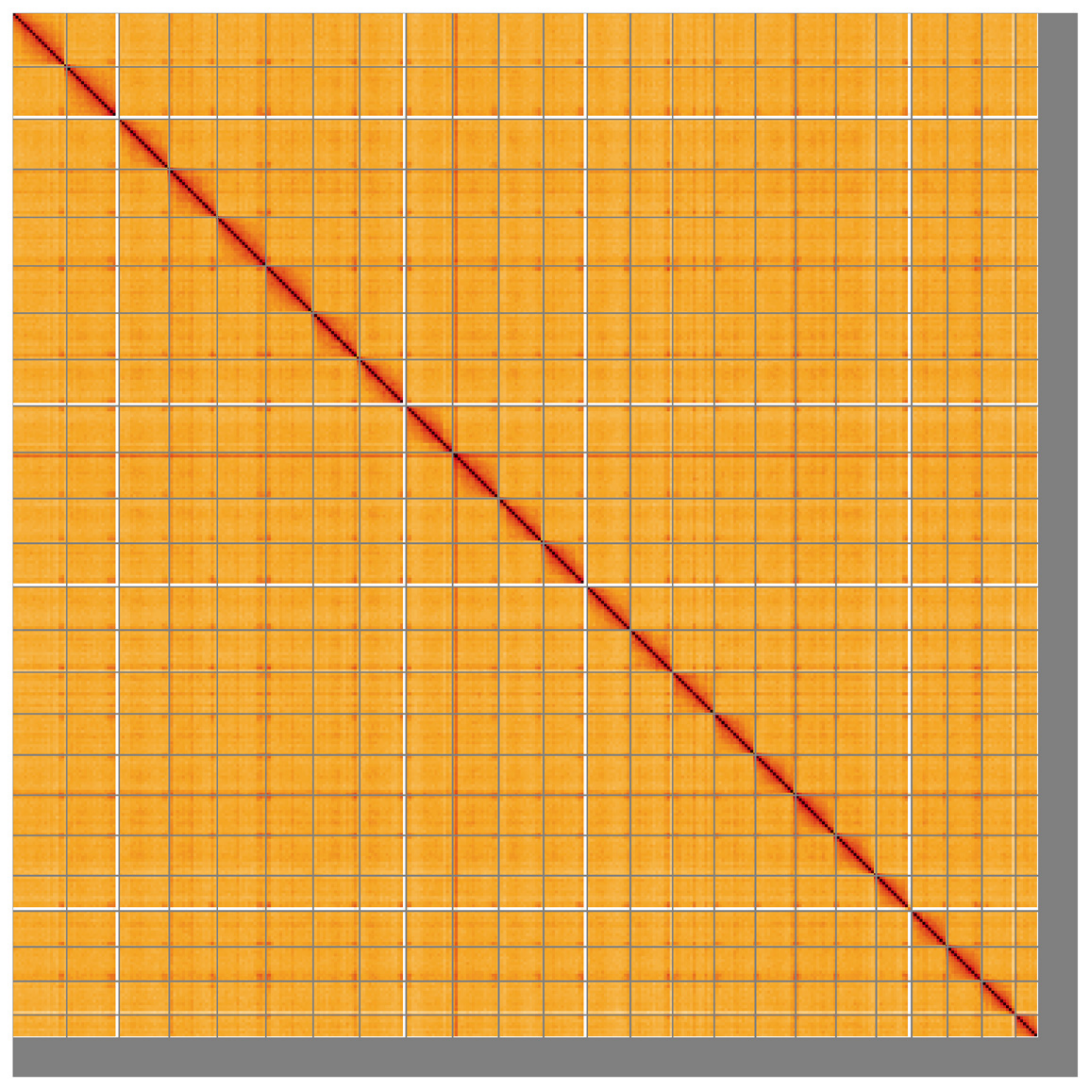
Genome assembly of *Scomber scombrus* fScoSco1.1: Hi-C contact map of the fScoSco1.1 assembly, visualised using HiGlass. Chromosomes are shown in order of size from left to right and top to bottom. An interactive version of this figure may be viewed at https://genome-note-higlass.tol.sanger.ac.uk/l/?d=VkyGE-XGQte5y0KeDM6FPg.

Manual assembly curation corrected 80 missing joins or mis-joins and 6 haplotypic duplications, reducing the scaffold number by 5.13%, and increasing the scaffold N50 by 2.29%. The final assembly has a total length of 764.10 Mb in 665 sequence scaffolds, with 1,489 gaps, and a scaffold N50 of 31.1 Mb ( Table 2). The snail plot in Figure 2 provides a summary of the assembly statistics, while the distribution of assembly scaffolds on GC proportion and coverage is shown in Figure 3. The cumulative assembly plot in Figure 4 shows curves for subsets of scaffolds assigned to different phyla. Most (95.81%) of the assembly sequence was assigned to 24 chromosomal-level scaffolds. Chromosome-scale scaffolds confirmed by the Hi-C data are named in order of size ( Figure 5; Table 3). While not fully phased, the assembly deposited is of one haplotype. Contigs corresponding to the second haplotype have also been deposited. The mitochondrial genome was also assembled and can be found as a contig within the multifasta file of the genome submission.

**Table 2.** Genome assembly data for *Scomber scombrus*, fScoSco1.1.

Genome assembly		
Assembly name	fScoSco1.1	
Assembly accession	GCA_963691925.1	
*Accession of alternate haplotype*	*GCA_963691945.1*	
Span (Mb)	764.10	
Number of contigs	2,155	
Contig N50 length (Mb)	1.5	
Number of scaffolds	665	
Scaffold N50 length (Mb)	31.1	
Longest scaffold (Mb)	38.75	
Assembly metrics *		*Benchmark*
Consensus quality (QV)	52.1	*≥ 50*
*k*-mer completeness	99.98%	*≥ 95%*
BUSCO **	C:98.1%[S:97.4%,D:0.7%], F:0.5%,M:1.4%,n:3,640	*C ≥ 95%*
Percentage of assembly mapped to chromosomes	95.81%	*≥ 95%*
Sex chromosomes	Not identified	*localised homologous pairs*
Organelles	Mitochondrial genome: 16.56 kb	*complete single alleles*

* Assembly metric benchmarks are adapted from column VGP-2020 of “Table 1: Proposed standards and metrics for defining genome assembly quality” from Rhie *et al.* (2021). ** BUSCO scores based on the actinopterygii_odb10 BUSCO set using version 5.4.3. C = complete [S = single copy, D = duplicated], F = fragmented, M = missing, n = number of orthologues in comparison. A full set of BUSCO scores is available at https://blobtoolkit.genomehubs.org/view/Scomber_scombrus/dataset/GCA_963691925.1/busco.

**Table 3.** Chromosomal pseudomolecules in the genome assembly of *Scomber scombrus*, fScoSco1.

INSDC accession	Name	Length (Mb)	GC%
OY829918.1	1	38.75	39.5
OY829919.1	2	37.13	39.5
OY829920.1	3	35.8	39.5
OY829921.1	4	34.6	39.5
OY829922.1	5	34.57	40.0
OY829923.1	6	33.76	40.0
OY829924.1	7	33.16	40.0
OY829925.1	8	33.14	39.5
OY829926.1	9	33.07	39.5
OY829927.1	10	32.93	40.0
OY829928.1	11	31.9	39.5
OY829929.1	12	31.1	39.5
OY829930.1	13	30.99	39.5
OY829931.1	14	30.23	39.5
OY829932.1	15	29.55	39.0
OY829933.1	16	29.28	39.5
OY829934.1	17	28.86	39.5
OY829935.1	18	28.78	40.0
OY829936.1	19	28.55	40.0
OY829937.1	20	25.45	40.0
OY829938.1	21	25.33	39.5
OY829939.1	22	24.62	40.0
OY829940.1	23	24.08	39.5
OY829941.1	24	16.57	40.0
OY829942.1	MT	0.02	47.0

The estimated Quality Value (QV) of the final assembly is 52.1 with *k*-mer completeness of 99.98%, and the assembly has a BUSCO v5.4.3 completeness of 98.1% (single = 97.4%, duplicated = 0.7%), using the actinopterygii_odb10 reference set ( *n* = 3,640).

Metadata for specimens, BOLD barcode results, spectra estimates, sequencing runs, contaminants and pre-curation assembly statistics are given at https://links.tol.sanger.ac.uk/species/13677.

## Methods

### Sample acquisition and DNA barcoding

A *Scomber scombrus* specimen (specimen ID MBA-220505-001A, ToLID fScoSco1) was collected from L4, English Channel, UK L4 (latitude 50.23, longitude –4.20) on 2022-05-05. The specimen was caught from the water column using a rod and line deployed from RV Sepia. Mitch Brenen, Kimberly Bird and Birthe Zaencker (Marine Biological Association) were in the collection team. The specimen was identified by Mitch Brenen (Marine Biological Association) based on gross morphology. The fish was first anesthetised and then overdosed using Aquased (2-phenoxyethanol). Destruction of the brain was used as a secondary method to ensure the animal was deceased before tissue sampling took place as in accordance with Schedule 1 methodology under the home office licence. Samples taken from the animal were preserved on dry ice.

The initial identification was verified by an additional DNA barcoding process according to the framework developed by Twyford *et al.* (2024). A small sample was dissected from the specimens and stored in ethanol, while the remaining parts of the specimen were shipped on dry ice to the Wellcome Sanger Institute (WSI). The tissue was lysed, the COI marker region was amplified by PCR, and amplicons were sequenced and compared to the BOLD database, confirming the species identification ( Crowley *et al.*, 2023). Following whole genome sequence generation, the relevant DNA barcode region was also used alongside the initial barcoding data for sample tracking at the WSI ( Twyford *et al.*, 2024). The standard operating procedures for Darwin Tree of Life barcoding have been deposited on protocols.io ( Beasley *et al.*, 2023).

### Nucleic acid extraction

The workflow for high molecular weight (HMW) DNA extraction at the Wellcome Sanger Institute (WSI) Tree of Life Core Laboratory includes a sequence of core procedures: sample preparation and homogenisation, DNA extraction, fragmentation and purification. Detailed protocols are available on protocols.io ( Denton *et al.*, 2023). The fScoSco1 sample was prepared by weighing and dissecting it on dry ice ( Jay *et al.*, 2023), and gill tissue was cryogenically disrupted using the Covaris cryoPREP ^®^ Automated Dry Pulverizer ( Narváez-Gómez *et al.*, 2023).

HMW DNA was extracted using the Automated MagAttract v2 protocol ( Oatley *et al.*, 2023a). DNA was sheared into an average fragment size of 12–20 kb in a Megaruptor 3 system ( Bates *et al.*, 2023). Sheared DNA was purified by solid-phase reversible immobilisation, using AMPure PB beads to eliminate shorter fragments and concentrate the DNA ( Oatley *et al.*, 2023b). The concentration of the sheared and purified DNA was assessed using a Nanodrop spectrophotometer and Qubit Fluorometer using the Qubit dsDNA High Sensitivity Assay kit. Fragment size distribution was evaluated by running the sample on the FemtoPulse system.

RNA was extracted from gill tissue of fScoSco1 in the Tree of Life Laboratory at the WSI using the RNA Extraction: Automated MagMax™ *mir*Vana protocol ( do Amaral *et al.*, 2023). The RNA concentration was assessed using a Nanodrop spectrophotometer and a Qubit Fluorometer using the Qubit RNA Broad-Range Assay kit. Analysis of the integrity of the RNA was done using the Agilent RNA 6000 Pico Kit and Eukaryotic Total RNA assay.

### Hi-C preparation

Gill tissue of the fScoSco1sample was processed at the WSI Scientific Operations core, using the Arima-HiC v2 kit. In brief, frozen tissue (stored at –80 °C) was fixed, and the DNA crosslinked using a TC buffer with 22% formaldehyde. After crosslinking, the tissue was homogenised using the Diagnocine Power Masher-II and BioMasher-II tubes and pestles. Following the kit manufacturer's instructions, crosslinked DNA was digested using a restriction enzyme master mix. The 5’-overhangs were then filled in and labelled with biotinylated nucleotides and proximally ligated. An overnight incubation was carried out for enzymes to digest remaining proteins and for crosslinks to reverse. A clean up was performed with SPRIselect beads prior to library preparation.

### Library preparation and sequencing

Library preparation and sequencing were performed at the WSI Scientific Operations core. Pacific Biosciences HiFi circular consensus DNA sequencing libraries were prepared using the PacBio Express Template Preparation Kit v2.0 (Pacific Biosciences, California, USA) as per the manufacturer's instructions. The kit includes the reagents required for removal of single-strand overhangs, DNA damage repair, end repair/A-tailing, adapter ligation, and nuclease treatment. Library preparation also included a library purification step using AMPure PB beads (Pacific Biosciences, California, USA) and size selection step to remove templates <3kb using AMPure PB modified SPRI. DNA concentration was quantified using the Qubit Fluorometer v2.0 and Qubit HS Assay Kit and the final library fragment size analysis was carried out using the Agilent Femto Pulse Automated Pulsed Field CE Instrument and gDNA 165kb gDNA and 55kb BAC analysis kit. Samples were sequenced using the Sequel IIe system (Pacific Biosciences, California, USA). The concentration of the library loaded onto the Sequel IIe was between 40–135 pM. The SMRT link software, a PacBio web-based end-to-end workflow manager, was used to set-up and monitor the run, as well as perform primary and secondary analysis of the data upon completion.

Poly(A) RNA-Seq libraries were constructed using the NEB Ultra II RNA Library Prep kit, following the manufacturer’s instructions. RNA sequencing was performed on the Illumina NovaSeq X instrument.

For Hi-C library preparation, DNA was fragmented to a size of 400 to 600 bp using a Covaris E220 sonicator. The DNA was then enriched, barcoded, and amplified using the NEBNext Ultra II DNA Library Prep Kit following manufacturers’ instructions. The Hi-C sequencing was performed using paired-end sequencing with a read length of 150 bp on an Illumina NovaSeq 6000 instrument.

### Genome assembly, curation and evaluation

***Assembly***

The HiFi reads were first assembled using Hifiasm ( Cheng *et al.*, 2021) with the --primary option. Haplotypic duplications were identified and removed using purge_dups ( Guan *et al.*, 2020). The Hi-C reads were mapped to the primary contigs using bwa-mem2 ( Vasimuddin *et al.*, 2019). The contigs were further scaffolded using the provided Hi-C data ( Rao *et al.*, 2014) in YaHS ( Zhou *et al.*, 2023) using the --break option. The scaffolded assemblies were evaluated using Gfastats ( Formenti *et al.*, 2022), BUSCO ( Manni *et al.*, 2021) and MERQURY.FK ( Rhie *et al.*, 2020).

The mitochondrial genome was assembled using MitoHiFi ( Uliano-Silva *et al.*, 2023), which runs MitoFinder ( Allio *et al.*, 2020) and uses these annotations to select the final mitochondrial contig and to ensure the general quality of the sequence.

***Assembly curation***

The assembly was decontaminated using the Assembly Screen for Cobionts and Contaminants (ASCC) pipeline (article in preparation). Flat files and maps used in curation were generated in TreeVal ( Pointon *et al.*, 2023). Manual curation was primarily conducted using PretextView ( Harry, 2022), with additional insights provided by JBrowse2 ( Diesh *et al.*, 2023) and HiGlass ( Kerpedjiev *et al.*, 2018). Scaffolds were visually inspected and corrected as described by Howe *et al.* (2021). Any identified contamination, missed joins, and mis-joins were corrected, and duplicate sequences were tagged and removed. The curation process is documented at https://gitlab.com/wtsi-grit/rapid-curation (article in preparation).

***Evaluation of the final assembly***

The final assembly was post-processed and evaluated with the three Nextflow ( Di Tommaso *et al.*, 2017) DSL2 pipelines “sanger-tol/readmapping” ( Surana *et al.*, 2023a), “sanger-tol/genomenote” ( Surana *et al.*, 2023b), and “sanger-tol/blobtoolkit” ( Muffato *et al.*, 2024). The pipeline sanger-tol/readmapping aligns the Hi-C reads with bwa-mem2 ( Vasimuddin *et al.*, 2019) and combines the alignment files with SAMtools ( Danecek *et al.*, 2021). The sanger-tol/genomenote pipeline transforms the Hi-C alignments into a contact map with BEDTools ( Quinlan & Hall, 2010) and the Cooler tool suite ( Abdennur & Mirny, 2020), which is then visualised with HiGlass ( Kerpedjiev *et al.*, 2018). It also provides statistics about the assembly with the NCBI datasets ( Sayers *et al.*, 2024) report, computes *k*-mer completeness and QV consensus quality values with FastK and MERQURY.FK, and a completeness assessment with BUSCO ( Manni *et al.*, 2021).

The sanger-tol/blobtoolkit pipeline is a Nextflow port of the previous Snakemake Blobtoolkit pipeline ( Challis *et al.*, 2020). It aligns the PacBio reads with SAMtools and minimap2 ( Li, 2018) and generates coverage tracks for regions of fixed size. In parallel, it queries the GoaT database ( Challis *et al.*, 2023) to identify all matching BUSCO lineages to run BUSCO ( Manni *et al.*, 2021). For the three domain-level BUSCO lineage, the pipeline aligns the BUSCO genes to the Uniprot Reference Proteomes database ( Bateman *et al.*, 2023) with DIAMOND ( Buchfink *et al.*, 2021) blastp. The genome is also split into chunks according to the density of the BUSCO genes from the closest taxonomically lineage, and each chunk is aligned to the Uniprot Reference Proteomes database with DIAMOND blastx. Genome sequences that have no hit are then chunked with seqtk and aligned to the NT database with blastn ( Altschul *et al.*, 1990). All those outputs are combined with the blobtools suite into a blobdir for visualisation.

The genome assembly and evaluation pipelines were developed using the nf-core tooling ( Ewels *et al.*, 2020), use MultiQC ( Ewels *et al.*, 2016), and make extensive use of the Conda package manager, the Bioconda initiative ( Grüning *et al.*, 2018), the Biocontainers infrastructure ( da Veiga Leprevost *et al.*, 2017), and the Docker ( Merkel, 2014) and Singularity ( Kurtzer *et al.*, 2017) containerisation solutions.

Table 4 contains a list of relevant software tool versions and sources.

**Table 4.** Software tools: versions and sources.

Software tool	Version	Source
BEDTools	2.30.0	https://github.com/arq5x/bedtools2
BLAST	2.14.0	ftp://ftp.ncbi.nlm.nih.gov/blast/executables/ blast+/
BlobToolKit	4.3.7	https://github.com/blobtoolkit/blobtoolkit
BUSCO	5.4.3 and 5.5.0	https://gitlab.com/ezlab/busco
bwa-mem2	2.2.1	https://github.com/bwa-mem2/bwa-mem2
Cooler	0.8.11	https://github.com/open2c/cooler
DIAMOND	2.1.8	https://github.com/bbuchfink/diamond
fasta _windows	0.2.4	https://github.com/tolkit/fasta_windows
FastK	427104ea91c78c3b8b8b49f1a7d6bbeaa869ba1c	https://github.com/thegenemyers/FASTK
Gfastats	1.3.6	https://github.com/vgl-hub/gfastats
GoaT CLI	0.2.5	https://github.com/genomehubs/goat-cli
Hifiasm	0.19.5-r587	https://github.com/chhylp123/hifiasm
HiGlass	44086069ee7d4d3f6f3f0012569789ec138f42b84aa44 357826c0b6753eb28de	https://github.com/higlass/higlass
Merqury.FK	d00d98157618f4e8d1a9190026b19b471055b22e	https://github.com/thegenemyers/ MERQURY.FK
MitoHiFi	3	https://github.com/marcelauliano/MitoHiFi
MultiQC	1.14, 1.17, and 1.18	https://github.com/MultiQC/MultiQC
NCBI Datasets	15.12.0	https://github.com/ncbi/datasets
Nextflow	23.04.0-5857	https://github.com/nextflow-io/nextflow
PretextView	0.2	https://github.com/sanger-tol/PretextView
purge_dups	1.2.5	https://github.com/dfguan/purge_dups
samtools	1.16.1, 1.17, and 1.18	https://github.com/samtools/samtools
sanger-tol/ ascc	-	https://github.com/sanger-tol/ascc
sanger-tol/ genomenote	1.1.1	https://github.com/sanger-tol/genomenote
sanger-tol/ readmapping	1.2.1	https://github.com/sanger-tol/readmapping
Seqtk	1.3	https://github.com/lh3/seqtk
Singularity	3.9.0	https://github.com/sylabs/singularity
TreeVal	1.0.0	https://github.com/sanger-tol/treeval
YaHS	1.2a.2	https://github.com/c-zhou/yahs

### Wellcome Sanger Institute – Legal and Governance

The materials that have contributed to this genome note have been supplied by a Darwin Tree of Life Partner. The submission of materials by a Darwin Tree of Life Partner is subject to the **‘Darwin Tree of Life Project Sampling Code of Practice’**, which can be found in full on the Darwin Tree of Life website here. By agreeing with and signing up to the Sampling Code of Practice, the Darwin Tree of Life Partner agrees they will meet the legal and ethical requirements and standards set out within this document in respect of all samples acquired for, and supplied to, the Darwin Tree of Life Project.

Further, the Wellcome Sanger Institute employs a process whereby due diligence is carried out proportionate to the nature of the materials themselves, and the circumstances under which they have been/are to be collected and provided for use. The purpose of this is to address and mitigate any potential legal and/or ethical implications of receipt and use of the materials as part of the research project, and to ensure that in doing so we align with best practice wherever possible. The overarching areas of consideration are:

•   Ethical review of provenance and sourcing of the material

•   Legality of collection, transfer and use (national and international)

Each transfer of samples is further undertaken according to a Research Collaboration Agreement or Material Transfer Agreement entered into by the Darwin Tree of Life Partner, Genome Research Limited (operating as the Wellcome Sanger Institute), and in some circumstances other Darwin Tree of Life collaborators.

## Data Availability

European Nucleotide Archive: *Scomber scombrus* (Atlantic mackerel). Accession number PRJEB69500; https://identifiers.org/ena.embl/PRJEB69500 ( Wellcome Sanger Institute, 2023). The genome sequence is released openly for reuse. The *Scomber scombrus* genome sequencing initiative is part of the Darwin Tree of Life (DToL) project. All raw sequence data and the assembly have been deposited in INSDC databases. The genome will be annotated using available RNA-Seq data and presented through the Ensembl pipeline at the European Bioinformatics Institute. Raw data and assembly accession identifiers are reported in Table 1 and Table 2.

## Author information

Members of the Marine Biological Association Genome Acquisition Lab are listed here: https://doi.org/10.5281/zenodo.8382513.

Members of the Darwin Tree of Life Barcoding collective are listed here: https://doi.org/10.5281/zenodo.12158331

Members of the Wellcome Sanger Institute Tree of Life Management, Samples and Laboratory team are listed here: https://doi.org/10.5281/zenodo.12162482.

Members of Wellcome Sanger Institute Scientific Operations: Sequencing Operations are listed here: https://doi.org/10.5281/zenodo.12165051.

Members of the Wellcome Sanger Institute Tree of Life Core Informatics team are listed here: https://doi.org/10.5281/zenodo.12160324.

Members of the Tree of Life Core Informatics collective are listed here: https://doi.org/10.5281/zenodo.12205391.

Members of the Darwin Tree of Life Consortium are listed here: https://doi.org/10.5281/zenodo.4783558.
