# Supplementary material for: The genome sequence of the Atlantic mackerel, Scomber scombrus Linnaeus, 1758
Source: Wellcome Open Res. 2024 Oct 17;9:610. [Version 1] doi: 10.12688/wellcomeopenres.23186.1 (PMC11558169; doi:10.12688/wellcomeopenres.23186.1)
Supplement: Peer review history

# Reviewer report: Reviewer response for version 1

**DOI:** 10.21956/wellcomeopenres.25536.r108918

**Published:** 2024-11-12

**Authors:** Emily Giles

**Affiliations:** 1 Universidad Austral de Chile, Valdivia, Los Ríos Region, Chile

The authors present the genome assembly of the Atlantic mackerel. They used PacBio Hifi sequencing with approximately 30X coverage. Hi-C mapping was used for the scaffolding. Approximately, 96% of the assembly was assigned to 24 chromosome level scaffolds. BUSCO scores indicate high levels of completeness and low levels of duplication. Overall, the methods are well described and complete. I have no major concerns with this genome report.

Are sufficient details of methods and materials provided to allow replication by others?

Yes

Is the rationale for creating the dataset(s) clearly described?

Yes

Are the datasets clearly presented in a useable and accessible format?

Yes

Are the protocols appropriate and is the work technically sound?

Yes

Reviewer Expertise:

Genomics, marine metazoa

I confirm that I have read this submission and believe that I have an appropriate level of expertise to confirm that it is of an acceptable scientific standard.

# Reviewer report: Reviewer response for version 1

**DOI:** 10.21956/wellcomeopenres.25536.r107532

**Published:** 2024-11-07

**Authors:** Elise Parey

**Affiliations:** 1 University College London, London, England, UK

The presented chromosome-scale genome assembly is of excellent quality and the methods appropriately described, both for wet-lab protocols (DNA extraction, purification, sequencing, Hi-C library preparation and sequencing) and the assembly pipelines. I do not have any further comment. I am convinced the genome will be a valuable resource for the field.

Are sufficient details of methods and materials provided to allow replication by others?

Yes

Is the rationale for creating the dataset(s) clearly described?

Yes

Are the datasets clearly presented in a useable and accessible format?

Yes

Are the protocols appropriate and is the work technically sound?

Yes

Reviewer Expertise:

Evolutionary Genomics

I confirm that I have read this submission and believe that I have an appropriate level of expertise to confirm that it is of an acceptable scientific standard.
